# Precision, Accuracy, and Observer Reliability of Computed Tomography‐Based Radiostereometric Analysis in Measuring Femoral Stem Migration In Vitro

**DOI:** 10.1002/jor.70146

**Published:** 2026-01-17

**Authors:** Vasileios Angelomenos, Maziar Mohaddes, Bita Shareghi, Henrik Malchau, Johan Nils Kärrholm, Raed Itayem

**Affiliations:** ^1^ Department of Orthopaedics Institute of Clinical Sciences, Sahlgrenska Academy University of Gothenburg Gothenburg Sweden; ^2^ Department of Hand Surgery Sahlgrenska University Hospital Gothenburg Sweden; ^3^ Department of Orthopaedics Skåne University Hospital Hässleholm Sweden; ^4^ Unit for Orthopaedics, Department of Clinical Sciences Lund University Lund Sweden; ^5^ Department of Orthopaedics Sahlgrenska University Hospital Gothenburg Sweden

**Keywords:** accuracy, computed tomography, CT, CT‐RSA, migration, precision, radiostereometric analysis, reliability, total hip arthroplasty

## Abstract

CT‐based radiostereometric analysis (CT‐RSA) is a method of measuring implant micromotion using low‐dose CT scans. We evaluated the precision, accuracy, and observer reliability of CT‐RSA for measuring femoral stem translation in THA in vitro. A cementless femoral stem was implanted in a synthetic femur mounted on a calibrated micrometer platform. Controlled translations were applied along each orthogonal axis across 5 series, generating 90 CT data sets. Only translational micromotions were imposed and analyzed. Precision was calculated from double examinations—repeated scans of the same displacement acquired in a different series—yielding 60 pairs. The femoral stem, head, and bone were segmented, the head's geometric center was defined, and translations were analyzed. Because the platform was fixed 90° to the femoral neck (CCD 135°), data were reported both on native axes, and after a 45° counterclockwise rotation to the conventional RSA frame. Precision was estimated from double examinations (SD × *t*), accuracy from RMSE versus micrometer readings (RMSE × *t*), and reliability from intraclass correlation coefficients (ICC) by two blinded observers. On native axes, precision ranged 0.06–0.08 mm and accuracy 0.09–0.13 mm. On transformed axes, precision ranged 0.06–0.07 mm and accuracy 0.03–0.14 mm. Intraobserver reliability was excellent (mean ICC 0.99 for all axes), as well as interobserver reliability (mean ICC 0.91–0.98). CT‐RSA achieved sub‐tenth‐millimeter precision with high repeatability and excellent observer agreement. These findings support CT‐RSA as a viable alternative for early migration measurement in THA research. Clinical validation with low‐dose protocols and predefined quality criteria remains warranted.

## 1 Introduction

Early implant migration within the first 2 years after hip arthroplasty is often used to predict future aseptic loosening of orthopedic implants [1, 2, 3, 4]. Radiostereometric analysis (RSA) has been the gold standard for measuring micromotions of orthopedic implants over the past few decades. However, RSA has some limitations [5] prompting the exploration of alternative methods.

Computed Tomography Micromotion Analysis (CTMA, Sectra, Sweden) is a CT‐based tool that measures implant micromotions without requiring bone or implant markers [5]. This method is commonly referred to in literature as CT‐RSA (Computed Tomography Radiostereometric Analysis or CT‐based Radiostereometric Analysis). The principles of CT‐RSA have been extensively studied for almost two decades [5, 6, 7, 8, 9, 10, 11, 12] and recent practical guidelines provide a concise overview of CT‑RSA methodology and best practices for study design, CT acquisition, analysis, and reporting across multiple joints [13, 14]. The technique is based on low‐dose CT scans, where threshold values are used to identify the bone and implant as rigid bodies. These rigid bodies constitute reference and moving segments used to calculate micromotions between repeated examinations.

Both in‐vitro and in‐vivo studies have demonstrated that the precision of CT‐RSA is comparable to traditional RSA methods [5, 7, 8, 9, 15, 16, 17, 18, 19, 20, 21]. Recent work has broadened the application of CT‐RSA beyond the hip. A systematic review including cervical disc, shoulder, hip, and knee arthroplasties reported high translational precision and accuracy with clinical precision in the acetabulum and proximal femur around 0.10–0.15 mm [22]. CT‐RSA techniques have also been applied to tibial and femoral components in total knee arthroplasty, midfoot joints, shoulder arthroplasty, and the sacroiliac joint, consistently demonstrating sub‐millimeter precision even when low‐dose CT protocols are used [23, 24, 25, 26, 27, 28]. Additionally, the applications of CT‐RSA have expanded even in smaller joints, such as the radiocarpal joint, showing comparable measuring performance to traditional marker‐based RSA [29, 30, 31]. These developments, together with practical CT‐RSA guidelines and an updated RSA/CT‐RSA reporting standard, illustrate that CT‐RSA has matured into a versatile migration analysis technique across multiple joints [13, 14]. Due to its marker‐free approach, reduced complexity in image acquisition, and comparable precision, CT‐RSA has been proposed as an alternative to marker‐based and model‐based RSA for measuring implant micromotions after total hip arthroplasty (THA) [5, 6, 7, 8, 10, 11, 12, 22, 32, 33].

The gradual introduction of new implants and techniques is crucial to minimize adverse events [34], which underscores the importance of reliable methods for monitoring implant migration. CT‐RSA could offer a reliable, marker‐free method to monitor early implant migration, thereby supporting the safe stepwise introduction and evaluation of new implants and surgical techniques.

Although several experimental studies have investigated the precision and accuracy of CT‐RSA [21, 35] for measuring implant migration, these studies typically involve a limited number of experiments, which may affect the generalizability of the findings. Furthermore, to date, no study has evaluated the intra‐ and interobserver reliability of CT‐RSA in an experimental setting specifically focused on femoral stems.

Our primary outcome measures were precision and accuracy of the CT‐RSA in measuring femoral stem micromotions along the *Y*‐axis (proximal–distal migration) due to its clinical relevance, while secondary outcome measures included precision and accuracy along the *X* (medial–lateral) and *Z* (anterior–posterior) orthogonal axis, as well as intra‐ and interobserver reliability of the method.

## 2 Materials and Methods

Study type: Basic science—phantom (in‐vitro) validation study.

Level of evidence: Not applicable (nonclinical).

**Figure 1:**
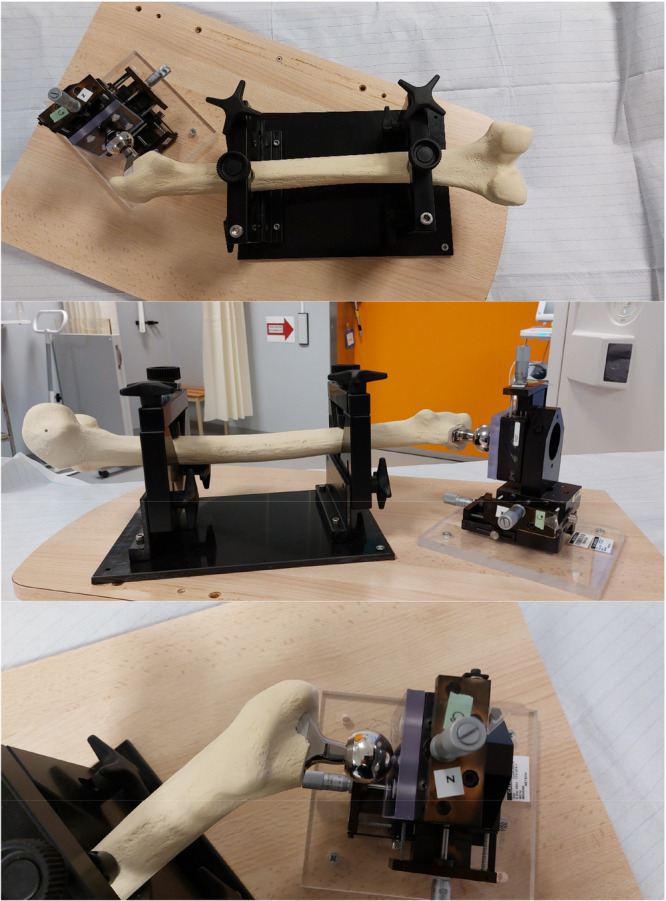
The experimental setup is shown here with the micrometer platform being mounted on the femoral caput at a 90° angle in relation to the femoral neck. The shaft of the femoral bone is secured by plastic screws to the platform. For further clarification, please see Figure 2 also.

**Figure 2:**
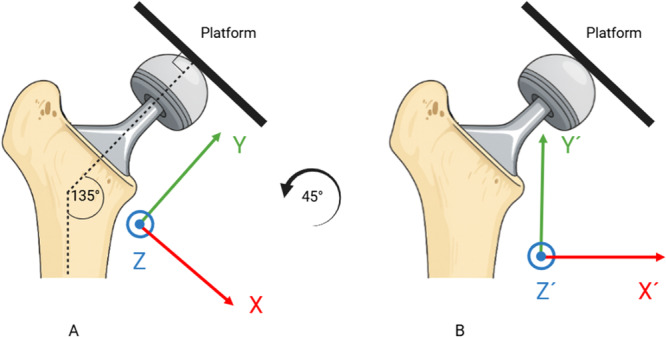
The figure illustrates the angle at which the platform is fixed to the femoral head, together with its relation to the femoral neck and the corresponding CCD angle. (A) The actual measurement axes (*X*, *Y*, *Z*) in relation to both the platform and the implant. After a 45° counterclockwise rotation, the transformed axes (*X*′, *Y*′, *Z*′) are displayed in (B).

A plastic model of a femoral bone (Sawbones, Pacific Research Laboratories Inc., Vashon Island, WA, USA) was prepared for implantation of a cementless femoral stem (Avenir Complete, Zimmer Biomet, Warsaw, Indiana, USA) allowing free movement of the stem within the prepared canal. The femoral shaft was mounted on a CT‐compatible platform using acrylic retainers and screws and the femoral stem was attached to a femoral head which in its turn was fixated with a screw and retainers to a calibrated *X*–*Y*–*Z* micrometer stage which was in its turn rigidly attached to the platform (Figure 1). The entire construct (femur and micrometer stage) was secured to the platform so that no relative motion could occur between the femur and the femoral stem other than the displacements applied through the micrometer screws. For technical reasons regarding stable fixation of the implant to the platform, the moving plate attached to the femoral head was fixated in a 90° angle in relation to the axis of the femoral neck and the neck itself had an angle of 135° in relation to the stem's central axis (Figures 1 and 2). The platform consisted of an *X*–*Y*–*Z* stage fitted with three spring‐loaded micrometers (linear accuracy of 2 µm, Parker Hannifin Corporation, Irwin, Pennsylvania, USA). The micrometer scales were divided into gradations of μm. Micromotions of known magnitude were performed by slightly adjusting the micrometer platform to simulate clinical positional variations and hence relative micromotions between the femoral stem and the femoral shaft. Because the micrometer stage was constructed to provide highly precise linear displacements but did not permit reproducible, isolated rotations about the stem axis, we restricted the experiment to translations. Accordingly, no controlled rotations were imposed, and all rotational degrees of freedom were set to zero during CT‑RSA registration and analysis.

Five series of CT scans were performed, each including six steps of micromotion (0, 25, 50, 75, 100, and 150 μm) per axis per series resulting in 90 CT scans in total. The stem was returned to the origin of the coordinate system (“point zero” *X*, *Y*, *Z*: 0, 0, 0) before the stepwise translation along each of the coordinate axes was commenced. The CT examinations were uploaded to the dedicated CTMA software (CTMA, Sectra, Linköping, Sweden) and analyzed.

All CT scans were performed in the same radiology department by experienced staff. The CT‐RSA analysis was executed by the same two authors (Vasileios Angelomenos and Bita Shareghi) that already have received training in the CTMA software. All radiological and statistical analyses were performed by investigators in our group (Vasileios Angelomenos and Bita Shareghi).

## 3 Transformation of the Coordinate System

The micrometer platform plate was fixed to the femoral head at 90° perpendicular to the stem's neck long axis. The implant's neck had a Caput‐Collum‐Diaphyseal (CCD) angle of 135°. For data extraction, the actual coordinate system was not aligned with the routine anatomical axes. Instead, it was offset by 45° clockwise about the anterior–posterior (*Z*) axis so that the *X*‐axis ran parallel to the platform plate and the *Y*‐axis parallel to the femoral neck axis in the anterior–posterior view to match the platform's axes of displacement. This alignment produced equal shifts in the *X*‐ and *Y*‐axes relative to the orientations typically used in published RSA and CT‐RSA studies, while the *Z*‐axis remained unchanged (Figure 2). To facilitate interpretation of the data in a research and clinical context we present precision and accuracy in both the actual coordinate system, as well the coordinate system typically used in previous RSA and CT‐RSA studies, which will be referred to as “transformed.” The actual coordinate system will be referred to as *X*–*Y*–*Z* and the transformed *X*´–*Y*´–*Z*´.

## 4 CT‐RSA

**Figure 3:**
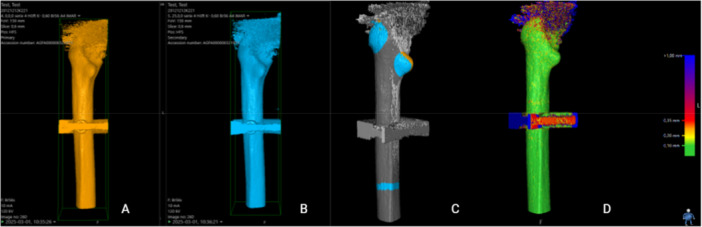
Segmentation and alignment of the femoral shaft. The shaft was segmented as the reference rigid body using a threshold of –800 HU (A and B). Alignment was performed by marking the major and minor trochanters and a ring on the distal femoral shaft including linea aspera (C). A color‐coded overlay confirmed correct alignment (D).

**Figure 4:**
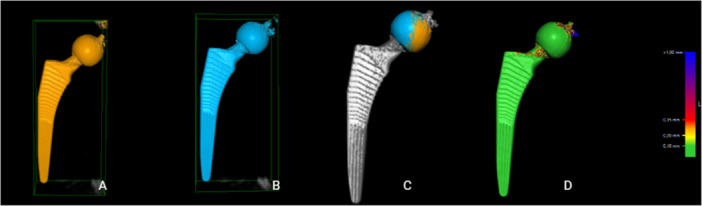
Segmentation and alignment of the femoral head as the moving rigid body. The head was segmented using a threshold of 2200 HU (A and B). Alignment was performed by marking the entire femoral head (C), with a color‐coded overlay verifying correct alignment (D).

**Figure 5:**
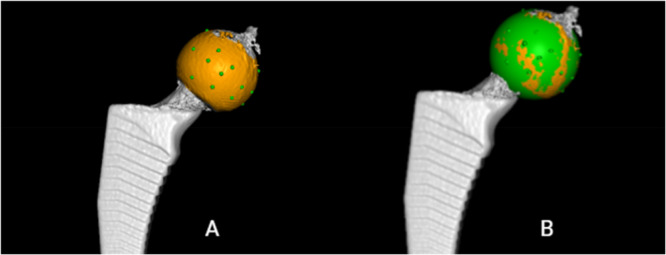
Definition of the geometric center of the femoral head. Multiple points (5–12, represented as green dots) were selected along the surface contour in the MPR view (A), defining a sphere whose center represented the femoral head's geometric center (B).

**Figure 6:**
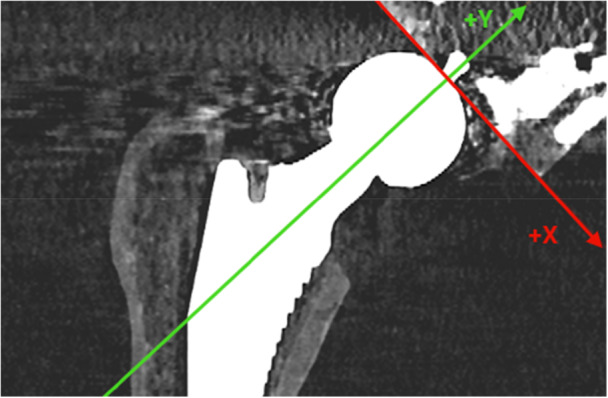
Adjustment of the coordinate system. The *X*‐axis was aligned parallel to the platform in the anteroposterior view, and the *Y*‐axis subsequently parallel to the implant neck. The *Z*‐axis remained unchanged. For illustration purposes, the lines representing the axes in the CT‐RSA software were made bolder and of different color. Consult, also, Figure 2 for further clarity.

All CT examinations were performed using a Somatom X.ceed scanner (Siemens Healthineers, Erlangen, Germany). A low‐dose CT protocol with Metal Artifact Reduction was applied with the following imaging parameters: tube current 15–100 mA (automatic), 100 kV, slice thickness 0.6 mm, increments 0.6 mm, pitch 0.8, rotation time 1 s, noise index 42.5, detector coverage 40 mm, reconstruction 0.6 mm. Prior to analysis, a protocol for measurement registration settings was established. To determine optimal registration parameters for the prosthesis and femoral Sawbone, six randomly selected scans were examined. A Hounsfield threshold of 2200 HU was set for implant segmentation, consistent with previous research [5, 32]. Due to the lower density of the Sawbone compared to normal femoral bone, the previously recommended segmentation threshold of 250 HU was insufficient. Consequently, a threshold of −800 HU was applied consistently for segmentation of the femoral shaft in all cases. The CT analysis process was done stepwise:

1. Position (*X*, *Y*, *Z*: 0, 0, 0) in each series was defined as “point zero” and the data set that corresponded to this position was used as a reference CT scan for that particular series. A CT‐scan data set that corresponded to simulated micromotion from the same series was uploaded into the CT‐RSA software.
2. The femoral shaft was segmented in both data sets as the reference rigid body using a threshold of −800 HU (Figure 3A,B) and the two data sets were aligned by marking the major and minor trochanters and a thin ring on the distal femoral shaft including linea aspera (Figure 3C).
3. A visual overlay of the femoral shaft was generated. The software provided a color‐coded overlay to assist in visually confirming correct alignment or determining if adjustments were needed to match the reference rigid body accurately (Figure 3D).
4. The femoral head, acting as the moving rigid body, was similarly segmented in both data sets using a threshold of 2200 HU (Figure 4A,B) and aligned by marking the femoral head in its entirety (Figure 4C).
5. A similar visual overlay was generated for the femoral head. Again, a color‐coded overlay assisted in verifying accurate alignment of the moving body (Figure 4D).
6. Since only translations were studied, rotations were set to zero.
7. The center of the femoral stem's head was defined as the reference point for the moving body. Multiple points (5–12) were selected along the surface contour of the femoral head using the software's crosshair tool in the multiplanar reconstruction (MPR) view (Figure 5A), thus defining a sphere with a center representing the geometric center of the femoral head (Figure 5B).
8. The coordinate system was adjusted so that the *X*‐axis was parallel to the platform in the anteroposterior view and the *Y*‐axis subsequently parallel to the neck of the implant (Figure 6).
9. Migration measurements were recorded in three degrees of freedom, corresponding to translations along the *X*‐, *Y*‐, and *Z*‐axis.

## 5 Statistics

The statistical analysis was performed using IBM SPSS version 28.0.0 software. All tests were two‐sided. The level of significance was set to *α* = 0.05. Pure mathematical analysis and transformation of the data were performed using Python v. 3.11 (Python Software Foundation, Wilmington, DE, USA).

### 5.1 Precision

Series 1 was used as the reference series. Position (*X*, *Y*, *Z*: 0, 0, 0) in each series was used as the reference CT scan on that particular series. Double examinations were defined as recordings that corresponded to identical amount of micromotion on a specific axis, but from another consecutive series, for example, Position 1/Series 1 and Position 1/Series 2 and so on, resulting in 60 pairs of double examinations. Any positional differences between two such examinations were regarded as random errors [36, 37]. The mean and standard deviation (SD) of the differences between the double examinations were calculated. The precision of the method was, determined using the SD of the differences multiplied by the critical value (*t*) obtained from the T‐table adjusted for the number of observations (Precision=SD×t, where “*t*” represents the value obtained in a *t*‐distribution table for a two‐sided 95% CI with “*n* −1” degrees of freedom, where “*n*” is the number of double examinations available, *n* = 60) [22, 38].

### 5.2 Accuracy

Accuracy of a measurement was defined as “the degree of closeness between a measured value and the true value and contains both random and systematic errors” [39]. Micrometer platform readings were considered as the true value and used as reference for calculating CT‐RSA method accuracy. To assess the accuracy of the CT‐RSA method, the mean signed differences (Bias), and the Root Mean Square Error (RMSE) of the differences between CT‐RSA measurements and known displacements on the micrometer platform were calculated. Accuracy was expressed by using RMSE adjusted using the critical *t*‐value for a two‐sided 95% confidence interval, as explained previously (Accuracy=SD×t) [22]. The RMSE was used since the difference between the readings on the micrometer platform and our CT data could be both positive and negative.

This approach to precision and accuracy is in line with the ISO 16087 RSA standard [38] and the recent guideline from the International Radiostereometry Society for RSA and CT‐RSA implant migration measurements [14].

### 5.3 Intra‐ and Interobserver Reliability

To assess intraobserver reliability, V.A.N. conducted the CT‐RSA analyses on all CT data sets twice, with a 14‐day interval, while unaware of the results from the first analysis. For interobserver reliability, B.S.H. carried out CT‐RSA analyses using the same protocol and was blinded to VAN's results. The intraobserver reliability was calculated using the intraclass correlation coefficient (ICC) [40] and a two‐way random, absolute agreement model. Likewise, this method was employed to assess the interobserver reliability by comparing the measurements made by authors V.A.N. and B.S.H. We report reliability only in the native (actual) axes. The transformed axes are merely a rotated coordinate system that mixes *X*′ and *Y*′ variance, where rotation leaves the 3D error unchanged, so per‐axis reliability after transformation adds no clinically interpretable value.

### 5.4 Transformation of the Data

The desired transformation was counterclockwise rotation of 45*°* (*θ* = +45*°*) of the *X*‐ and *Y*‐axis about the *Z*‐axis (Figure 2). A rotation matrix was established, and every measurement was transformed according to the equation: M′=Rz(θ)×M, where *M*′ is the transformed measurement, $Rz\;(\theta )=\left[\left.\begin{array}{ccc} \cos\theta & -\sin\theta & 0\\ \sin\theta & \cos\theta & 0\\ 0 & 0 & 1 \end{array}\right]\right.$ the rotation matrix, and *M* the actual measurement. Then precision and accuracy were recalculated as described above.

## 6 Results

### 6.1 Precision

On the actual axes, the precision of the CT‐RSA method for translations along the *X*‐ (medial–lateral), *Y*‐ (proximal–distal), and *Z*‐axis (anterior–posterior) was 0.08, 0.06, and 0.07 mm, respectively.

On the transformed axes, the precision was 0.06 mm on the *X*′‐ (medial–lateral) and *Y*′‐axis (proximal–distal) and 0.07 mm on the *Z*′‐axis (anterior–posterior).

### 6.2 Accuracy

On the actual axes, the accuracy of the CT‐RSA was 0.09 mm for the *X*‐axis (medial–lateral), 0.13 mm for the *Y*‐axis (proximal–distal), and 0.11 mm for the *Z*‐axis (anterior–posterior).

On the transformed axes, the accuracy was 0.03 mm for the *X*′‐axis (medial–lateral), 0.14 mm for the *Y*′‐axis (proximal–distal), and 0.11 mm for the *Z*′‐axis (anterior–posterior).

### 6.3 Intra‐ and Interobserver Reliability

The intraobserver reliability, ICC (95% CI) was 0.99 (0.98–0.99) on *X*‐ (medial–lateral), and *Y*‐axis (proximal–distal), and 0.99 (0.97–0.99) on *Z*‐axis (anterior–posterior).

The interobserver reliability, ICC (95% CI) for the *X*‐axis (medial–lateral), was 0.91 (0.74–0.97), for the *Y*‐axis (proximal–distal) 0.98 (0.93–0.99), and for the *Z*‐axis (anterior–posterior) 0.96 (0.88–0.99).

Detailed values of all outcome measures are summarized in Tables 1, 2, 3.

**Table 1:** Precision estimates of the CT‐RSA method based on 60 double examinations shown along the three orthogonal axes, where *X*, *Y*, and *Z* represent the original measurement axes for translation, and *X*′, *Y*′, and *Z*′ the transformed axes.

Precision estimates							
Actual data				Transformed			
Axis	Mean	SD	Precision	Axis	Mean	SD	Precision
*X*	0.015	0.041	0.08	*X*′	0.001	0.031	0.06
*Y*	0.001	0.030	0.06	*Y*′	0.011	0.031	0.06
*Z*	−0.014	0.034	0.07	*Z*′	−0.014	0.034	0.07

*Note:* Mean values and standard deviations (SD) of the differences between the double examinations are presented together with corresponding precision (SD ×* t*) in millimeters.

**Table 2:** Accuracy estimates of the CT‐RSA method based on 90 paired examinations shown along the three orthogonal axes, where *X*, *Y*, and *Z* represent the original measurement axes for translation, and *X*′, *Y*′, and *Z*′ represent the transformed axes.

Accuracy estimates							
Actual data				Transformed			
Axis	Bias	RMSE	Accuracy	Axis	Bias	RMSE	Accuracy
*X*	0.011	0.05	0.09	*X*′	0.002	0.02	0.03
*Y*	0.008	0.07	0.13	*Y*′	0.013	0.07	0.14
*Z*	−0.035	0.06	0.11	*Z*′	−0.035	0.06	0.11

*Note:* Mean signed differences (Bias) and Root Mean Square Error (RMSE) of the differences between CT‐RSA and micrometer platform readings are presented together with corresponding accuracy (RMS × *t*) in millimeters.

**Table 3:** Intra‐ and interobserver reliability of the CT‐RSA method along the three orthogonal axes presented as mean ICC, where *X*, *Y*, and *Z* represent the original measurement axes for translation along with the 95% confidence intervals (CI).

	Intraobserver reliability		Interobserver reliability	
Axis	Mean	95% CI	Mean	95% CI
*X*	0.99	0.98–0.99	0.91	0.74–0.97
*Y*	0.99	0.98–0.99	0.98	0.93–0.99
*Z*	0.99	0.97–0.99	0.96	0.88–0.99

## 7 Radiation Dose

The mean effective radiation dose for the scans used in the CT‐RSA analysis was estimated to be 0.08 mSv/scan.

## 8 Discussion

Our study demonstrated that the CT‐RSA method has a precision of 0.06 mm along the *Y*′‐axis (proximal–distal translation), which aligns with findings reported in prior literature [22]. The secondary outcomes evaluated included translations along the *X*′ (medial–lateral) and *Z*′ axes (anterior–posterior), where the method exhibited similar precisions of 0.06 and 0.07 mm, respectively. Precision values for the CT‐RSA method in this study were better than previously reported by the same research group [5], as well as other researchers [16] who reported similar precision ranges for CT‐RSA in evaluating implant micromotion following THA in clinical scenarios. These differences, though, should be expected as these studies were clinical and by definition face more challenges in image acquisition, including movement artifacts and soft tissue interference, potentially compromising the image quality compared to a plastic phantom model. Nevertheless, the precision of CT‐RSA in the current study is still higher on all axes compared to previous in vitro studies [19, 35] that reported a mean precision of 0.13 mm for translations of the femoral stem [22].

The accuracy of the CT‐RSA in the current study was better on the *X*′‐ (medial–lateral) and *Y*′‐axis (proximal–distal) compared to previous experimental studies [21, 35]. On the *Z*′‐axis (anterior–posterior) the accuracy was worse than one previous study by Clarke et al. [35] (0.043 mm), but better than in the study by Scheerlinck et al. (0.163 mm) [21], though a different type of phantom, software and mathematical approach was used in these studies to calculate accuracy. Namely, Clarke et al. [35] used a custom‐made phantom model and tantalum beads, and a nonimplant‐migration‐dedicated software (Mimics v17.0, Materialise, Leuven, Belgium) in the segmentation and measurement process. Scheerlinck et al. [21] used a marker‐free approach on a cadaver specimen, but a different mathematical approach using the mean signed differences (bias) between examinations was employed, although even RMSE × *t* estimates were also presented, which we have used as a comparison in order to have a uniform reporting. We have no other direct explanation regarding these differences, but the use of another experimental setup, software, fewer measurements than in the current study that could potentially hide a bias and the use of tantalum beads as reference points and a different mathematical approach to measuring accuracy in one of these studies [21] could influence the results.

The intra‐ and interobserver reliability in this study were excellent, which is consistent with prior studies [32, 41]. Our findings further confirm the intra‐ and interobserver reliability of this method, underscoring the robustness and reproducibility of CT‐RSA measurements, as demonstrated in other implant micromotion studies utilizing CT‐RSA [32, 41].

From a clinical perspective, the relevance of the sub‐tenth‐millimeter translational precision observed in our phantom model needs to be considered in relation to established RSA thresholds for unacceptable migration. Meta‐analyses of conventional RSA studies have suggested that proximal cup migration up to 0.2 mm at 2 years and stem subsidence below 0.15 mm are associated with low (< 5%) 10‐year revision rates, whereas larger early migrations are linked to substantially higher revision risks [42]. The translational precision of CT‐RSA in our in‐vitro setup (≈0.06–0.07 mm along the clinically relevant axes) is therefore well below the magnitude of migration considered clinically important. Even though clinical CT‐RSA studies generally report somewhat lower precision than phantom experiments, typical clinical precision in the range of 0.10–0.15 mm in the hip [22, 24, 28] remains adequate to distinguish safe from at‐risk implants at the group level. Thus, while sub‐tenth‐millimeter precision may exceed what is strictly required for predicting aseptic loosening, it provides a wide safety margin and supports the use of CT‐RSA as a sensitive tool for early migration surveillance.

Despite the promising findings, this study carries several limitations. Primarily, being an in‐vitro analysis based on a synthetic femur without soft tissue, our experimental setup, although precisely controlled, does not entirely reflect the clinical complexities associated with interindividual bone heterogeneity, soft tissue interference, and radiation scatter, artifacts or patient motion during image acquisition, factors that may reduce precision and accuracy in vivo compared with the present phantom results. Additionally, the accuracy assessment was based on micrometer measurements considered as the “gold standard,” yet even minor inaccuracies inherent in mechanical platforms could potentially influence the measured outcomes. Moreover, due to technical challenges concerning the fixation of the phantom to the micrometer platform, we chose to perform the migration analysis in the platform's/phantom's geometrical plane and then transform the data. Purely mathematically, the transformation is not expected to alter the data or the total 3D error; however, the transformed coordinate system remains a theoretical construct. While it aligns with the reference frame commonly used in prior RSA and CT‐RSA studies, it represents a derived mathematical interpretation rather than a direct physical measurement. Additionally, we deliberately restricted the experiment to pure translations and that could be consider a limitation of this study. The micrometer platform was specifically designed to apply highly controlled linear displacements along three orthogonal axes, whereas introducing small, well‐defined rotations around the femoral stem axis would have required a substantially more complex mechanical setup and would have increased uncertainty in the “true” imposed motion. As a result, rotational precision and accuracy—although clinically relevant for some stem designs—were not evaluated in this study and should be addressed in future CT‐RSA validation work. Another limitation relates to the radiological exposure; although CT‐RSA uses a relatively low‐dose radiation protocol, repeated CT scans in clinical practice remain a concern that necessitates careful consideration to potentially lower the mean effective radiation dose used.

Future phantom and clinical CT‐RSA studies should include rotational data to complement the present translational evaluation. Future research should focus on further clinical validation of these findings, including comparative in‐vivo studies involving large patient cohorts and longitudinal follow‐ups to evaluate the long‐term reliability and clinical relevance of CT‐RSA in predicting implant loosening. Furthermore, assessing CT‐RSA performance across various implant designs, fixation techniques, bone qualities, and over longer periods (accounting even for bone remodeling issues) could enhance the generalizability of this measurement technique. Additional comparative studies investigating radiation exposure in different CT‐imaging modalities and protocols may help optimize the balance between diagnostic efficacy and patient safety. Finally, developing automated [33] or semi‐automated software processes could further minimize observer variability and enhance the clinical practicality of CT‐RSA as a routine research tool for postoperative monitoring of THA implants.

## 9 Conclusion

In vitro, CT‐RSA measured femoral stem translations with high precision and accuracy, and excellent intra‐ and interobserver reliability. According to our data, CT‐RSA can reliably measure femoral stem translations in a controlled environment. Clinical use should include standardized protocols, analyst training, and dose optimization. Under these conditions, CT‐RSA can be used for implant migration surveillance. Nevertheless, further validation of the method in vivo is warranted to confirm performance in clinical settings.

## Author Contributions

Maziar Mohaddes, Raed Itayem, Henrik Malchau, Johan Nils Kärrholm, and Vasileios Angelomenos conceptualized the study. All authors were involved in the study design. Bita Shareghi and Vasileios Angelomenos retrieved and prepared the data. Vasileios Angelomenos performed the statistical analyses. Vasileios Angelomenos wrote the manuscript, and all authors contributed with notable critical appraisal of the text and approved the final version.

## Ethics Statement

The authors have nothing to report.

## Conflicts of Interest

The authors declare no conflicts of interest.

## Data Availability

Sharing of data is available upon request.
